# Viability of reducing plastic waste disposal by utilization in hot mix asphalt: a multicriteria analysis with cost considerations

**DOI:** 10.1007/s11356-026-38290-x

**Published:** 2026-10-09

**Authors:** Adnan Qadir, Uneb Gazder

**Affiliations:** 1 Department of Urban and Infrastructure Engineering, NED University of Engineering and Technology, Karachi, 75270 Pakistan; 2 Department of Civil Engineering, School of Engineering, Monash University Malaysia, Jalan Lagoon Selatan, 47500 Bandar Sunway, Selangor Malaysia; 3 Monash Climate-Resilient Infrastructure Research Hub (M-CRInfra), School of Engineering, Monash University Malaysia, Jalan Lagoon Selatan, 47500 Bandar Sunway, Selangor Malaysia

**Keywords:** Recycled plastic waste aggregate, Marshall stability, Sustainable pavement, Polyethylene bag waste, Optimal replacement content, Pakistan

## Abstract

Plastic waste is a non-biodegradable material which is extensively used in many applications, creating a critical environmental challenge of its disposal. Therefore, its utilization in large quantities as a recycled product in road infrastructure will mitigate ill effects occurring because of its dumping. The aim of this study was to analyze the impacts of adding recycled plastic waste aggregate (RPWA) in hot mix asphalt (HMA) mixes using a wide range of variables by employing the Marshall mix design procedure in the context of Pakistan. A data-driven framework is employed with a novel multivariate performance score function. Incorporation of RPWA, sourced from LDPE polyethylene bags, at the content of 2.5% by weight (~ 6% by volume) was found to provide the best strength while fulfilling criteria of other key parameters. The mix performance degrades steeply and significantly beyond 2.5% but remains under acceptable design ranges till 10%. Based on these results, RPWA can be used to enhance pavement performance with strict quality control on batching. The findings of this study pave the way towards a sustainable pathway for high-volume disposal of low-value plastic film waste with applications in low to medium-traffic roads. Investigations with smaller increments in RPWA content, field testing, and lifecycle cost analysis (LCCA) are recommended for future research directions. The findings of this study highlight the impacts of using RPWA in road infrastructure providing ways to reduce plastic pollution through optimum design of pavements. However, they are based on Marshall laboratory testing and require validation through durability, moisture, rutting, fatigue, and field studies before implementation.

## Introduction

Plastics contain various chemical additives, such as phthalates and bisphenol A, which can leach into the environment through soil and water over time, posing risks to both the existence of wildlife and human health (Tumu et al. 2023). On the other hand, large plastic debris and microplastics also have the risk of endangering wildlife through ingestion and entanglement (Blettler and Mitchell 2021).

Current waste management systems are overwhelmed by the sheer volume and complex nature of plastic waste. Global plastic production and waste generation are rising relentlessly, expected to be triple its amount from last decade by the end of 2060 (Lebreton and Andrady 2019). This growth rate outpaces the capacity of existing waste management and recycling infrastructure. Globally, only a small fraction, ~ 9% of plastic waste is successfully recycled while the rest ends up incinerated, landfilled, or leaked into the environment (Thiounn and Smith 2020). Many regions, especially developing nations like Pakistan, lack the necessary collection, sorting, and processing facilities to manage plastics effectively (Browning et al. 2021). This leads to high rates of mismanaged waste and open dumping.

Another issue is the existence of numerous types of plastics and multi-material products which make sorting and recycling technically difficult and often uneconomical (de Mello Soares et al. 2022). Consequently, it leads to lower-quality recycled materials. Furthermore, huge volumes of non-recycled plastic are sent to landfills, consuming valuable land and leading to adverse environmental impacts in the form of release of methane gas during decomposition in anaerobic conditions, and groundwater contamination through the mixing of toxic leachate into groundwater (Wojnowska-Baryła et al. 2022). Incineration, an alternative to landfill disposal systems, reduces volume of plastic on one hand but contributes to air pollution in the form of greenhouse gases and other toxic air pollutants (Cai et al. 2023).

The global rise in plastic production and waste creates a direct, twofold responsibility for the construction industry. Firstly, to address the massive amount of plastic it uses and, secondly, to leverage its scale in absorbing post-consumer plastic waste. The construction industry is a major consumer of global plastic production with amounts reaching up to 20% (Royal Institution of Chartered Surveyors 2023), consequently playing a significant role in the rise of global non-degradable plastic waste totals. In some cases, the building and construction sector is the second-largest market for plastics (Akovali et al. 2005), only second to packaging. The commonly used applications of plastics in construction industry include PVC piping, insulation, window frames, and waterproofing membranes (Gupta et al. 2024). Moreover, a significant portion of the plastic waste on construction sites also comes from single-use packaging, such as films, wraps, straps, which are used to protect materials (Stemberger and Pothireddy 2022). Most of this packaging is difficult to recycle and often ends up in landfills or the environment.

The plastic component of construction and demolition (C&D) waste is often a mix of different polymer types which may be contaminated (Santos et al. 2024). This results in low recycling and high disposal rates for plastics, compared to other C&D materials like concrete and metal. All these issues underscore the need for the construction sector to transition to a circular economy model. It is also worth mentioning that the production of virgin plastic, like other traditional construction materials, is energy-intensive and a major source of embodied carbon in the form of CO2 emissions (Ganesan et al. 2023). Using recycled plastic significantly reduces this environmental footprint by reducing the need for production of construction materials while disposing of plastic safely. Furthermore, use of recycled plastic in the construction industry reduces the demand for new fossil fuels and decreases the need for new material extraction. This approach will minimize damage from mining and quarrying to the ecosystem while conserving scarce natural resources. Leveraging plastic waste in construction diverts millions of tons of material from landfills protecting the ecosystem from the harmful impacts of plastic, which have been discussed above.

Given the size of the sector, the construction industry can act as a massive end market for hard-to-recycle post-consumer plastic waste (Yen et al. 2020). Even a small shift of industry towards recycled plastic can absorb a significant amount of the world’s annual plastic waste, making it a critical player in global sustainability efforts. This step is crucial in promoting a circular economy and shifts the viewpoint related to plastic waste to a resource rather than a liability. To this end, recycled plastics are being successfully incorporated into various building products, including plastic lumber, composite roofing tiles, non-structural bricks and blocks, asphalt additives, insulation and acoustic panels (Lamba et al. 2022).

Hot mix asphalt (HMA) is extensively used worldwide, including in countries with large road networks like India and Iraq. Its widespread adoption is due to its proven performance and the availability of established production and laying techniques (Bellary and Kadli 2023). It provides a durable road surface capable of withstanding heavy traffic loads and adverse weather conditions. This durability is crucial for maintaining road quality and reducing the frequency of maintenance and rehabilitation works (Sol-Sánchez et al. 2016). The main reason for its use in roads is its excellent mechanical properties, including high resistance to rutting and fatigue cracking, which are essential for the longevity of road pavements (Alo and Oyedepo 2024). Apart from roads, HMA is extensively used worldwide for constructing airport runways and parking lots (Chong et al. 2014).

The rise in road construction and maintenance projects has led to a higher demand for the relevant construction materials including fresh aggregates and HMA (Dannalie and Nataadmadja 2021). This is particularly evident in countries with extensive road networks, such as Malaysia and the United States, requiring frequent maintenance (Global Market Insights, Inc. 2025).

The production of HMA involves high energy consumption and significant emissions of greenhouse gases (GHGs) due to the high temperatures required for mixing and laying (Whaley et al. 2017). The emissions of volatile organic compounds (VOCs) and GHGs pose health risks to workers and contribute to environmental pollution (Sharma et al. 2020). This has led to a growing emphasis on sustainability in road construction. Efforts are being made to reduce the environmental impact of HMA by incorporating recycled materials and developing alternative technologies like warm mix asphalt (WMA) (Shah et al. 2024).

There are several key parameters which influence the performance of HMA. The maximum load carrying capacity and its resistance to deformation is assessed through Marshall stability (Awuah and Thom 2023). The flexibility of asphalt mix is measured in terms of flow value which provides insights into the pavement flexibility and resistance to common failures, such as rutting (Awuah and Thom 2023).

The ease with which the HMA can be compacted during construction affects its final density, consequently, the performance of the pavement (Komba et al. 2019). In this regard, the voids in the HMA mixture play a vital role. The percentage of air spaces within the compacted HMA is essential for preventing moisture damage and ensuring durability (Krishna Rao and Sravana 2023). Moreover, the volume of void spaces between the aggregate particles in a compacted mix is also required to be adequate in order to accommodate enough binder for durability and resistance to deformation (Islam et al. 2019). Apart from that, the percentage of VMA filled with asphalt binder also helps in balancing the mix to prevent both rutting and cracking (Krishna Rao and Sravana 2023). An important element of HMA mix is the aggregate whose size distribution affects the compactability, stability, and resistance to deformation of the HMA (Gong et al. 2019). In this study, the aggregates were sourced from the local quarries and used as per national highway standards which is further explained in the succeeding sections. Another selection from local standards is the asphalt binder whose amount in the mix affects durability, flexibility, and resistance to various distresses such as rutting and cracking (Bastidas-Martínez and Rondon-Quintana 2024). Moreover, binder viscosity and stiffness are crucial properties for determining the mix’s resistance to deformation and cracking under different temperature conditions (Gong et al. 2019).

The global environmental crisis from plastic waste is primarily driven by its non-biodegradability and the substantial challenges in its disposal. It is mainly due to the durability and chemical inertness of plastic, which are also the characteristics that make plastic so versatile (Harvey 2005). Most conventional plastics are not biodegradable, as they do not decompose naturally by microorganisms within a reasonable timeframe. They can persist in the environment for many decades, resulting in their continuous accumulation in ecosystems. Over the course of time, plastic waste breaks down into increasingly smaller fragments called microplastics (< 5 mm) and nanoplastics contaminating soil, water, and air (Yee et al. 2021).

There is a body of research available which has investigated the use of different types of plastic waste in bitumen as well as in HMA (Masad et al. 2020). This includes the use of high density polypropylene, high-density polyethylene (HDPE), and low-density polyethylene (LDPE). All three types of plastics have shown good results with increasing fatigue resistance when added to bitumen in low amounts (0–6%) (Ghani et al. 2022). In terms of durability of modified bitumen mixtures, the addition of HDPE and LDPE is to reported enhance rutting, and resilient modulus (MR) performance with an increase of 19 and 9%, respectively (Dalhat and Al-Abdul Wahhab 2017). Other areas of using HDPE and LDPE in asphalt are as replacement of aggregates, which is also targeted in the present study. The early results show an increase in asphalt compactness (less air voids and higher density) with replacements of aggregates up to 25% by HDPE and LDPE (Ullah et al. 2021). The use of LDPE-modified bitumen in the mix has also shown higher benefits in terms of fatigue (57% higher fatigue life) and costs (10% reduction) compared to other modifications including crumb rubber as well as traditional binders in India (Singh and Gupta 2024). This finding was further reinforced by another study based on SHRP (Strategic Highway Research Program) method which also established that LDPE are more suitable for low-temperature site conditions (Liang et al. 2021) . In terms of high-temperature conditions, mixtures with LDPE-modified bitumen showed higher performance than traditional mixtures, when optimally blended with an elastomer such as styrene butadiene styrene (SBS) (Nisar and Mir 2023). The LDPE-modified bitumen has also been observed to maintain the positive correlation with workability similar to traditional mixtures (Yu et al. 2024).

Use of plastic waste in HMA is a promising and actively researched application that offers significant environmental and engineering benefits. While the environmental benefits have already been mentioned above, it can lead to decreasing the demand for virgin asphalt binder or virgin mineral aggregates, depending upon its application. Given the nature of highway projects and their resource requirements, the benefits stemming from plastic use would be large-scale, valuable, and long-term. Furthermore, it has the potential to create a new class of construction material with potential of increased durability, cost-effectiveness, or sustainability compared to conventional asphalt mix.

Based upon these points, previous studies have explored the use of recycled plastic in asphalt, establishing a foundation for its feasibility. These efforts can be mainly categorized in two aspects: the use of plastic as a binder modifier and its use as replacement of aggregates. The latter is termed as recycled plastic waste aggregates (RPWA) in this study. The modification of asphalt with recycled plastics has been reported to increase the rutting resistance of asphalt, at high temperatures (Joni and AL-Rubaie 2022), and decrease consistency of asphalt binder (Abuaddous et al. 2021). On the other hand, use of recycled plastic as aggregate replacement is found to be cost-effective as compared to its application as binder modification (White and Hall 2022). The replacement application of recycled plastic is reported to improve the adhesion between the bitumen and aggregates (Amas and Khurshid 2025). In addition to that, some studies show improved overall mechanical properties like Marshall Stability and reduced rut depth (Khan et al. 2019).

Despite these efforts, certain gaps still exist in literature. They are as follows:

- A comprehensive multi-parameter analysis linking the percentage of RPWA to a wide range of properties which are critical for Marshall design procedure
- A rigorous statistical evaluation to definitively quantify the impact of RPWA content on each parameter of Marshall process
- Performance-based optimization to determine a practical acceptable range for RPWA content
- Studies related to analyzing the impacts and optimizing the range of RPWA with locally sourced materials for Pakistan

In order to contribute towards filling these gaps, this study aims to analyze the impacts of RPWA content on a wide range of Marshall properties with the aim to determine the practically acceptable range for it. The aim is achieved through the following objectives:

1. To produce and test HMA samples modified with different percentages (0 to 16%) of RPWA
2. To evaluate the impact of RPWA content on key engineering properties for the Marshall process
3. To perform statistical analysis, using ANOVA, to determine the significance of plastic content’s influence on these properties
4. To establish an optimal and acceptable range for recycled plastic content based on a proposed composite performance score
5. To provide a preliminary assessment of the preparation cost for the modified mix with RPWA

Thus, the manuscript attempts to make a novel contribution with a robust and pragmatic multicriteria scoring method which could be implemented in design without the need for complex formulations or models. The score is based upon a set of Marshall method features which could be conveniently collected through simpler lab tests and directly addresses the design concerns. The scope of current research is based on locally sourced aggregates and focuses on the use of LDPE as the modification. Moreover, it employs Marshall Mix Design method in experimental procedures.

This research offers a sustainable waste management solution and a potential enhancement to pavement performance. LDPE, used in this research for modification, was sourced from shopping bags also referred to as polythene bags. Its selection is due to the issues related to management in disposal in the local context of Pakistan. Approximately 10 million plastic bags are discarded daily as waste. Only 10–15% are disposed of in dustbins, with the remainder ending up in drains, sewage channels, and open spaces. It is estimated that 80% of the country’s drains are obstructed by polyethylene bags, exacerbating health risks in a nation already vulnerable to severe hazards (United Nations Development Programme (UNDP) 2021). Blocked drains cause sewage to contaminate drinking water supplies, contributing to the spread of waterborne diseases. A recent Gallup Pakistan survey indicates that plastic bag usage is nearly equal in urban and rural areas, with 75% of people using plastic bags, compared to just 6% using paper bags, 17% using cloth bags, and only 2% using baskets. Hence, LDPE, extracted from these bags, was selected due to dire issues related to it (Gallup and Gilani Pakistan 2023).

The remainder of the paper is arranged as follows. The section describes materials and methods; the section presents the results, and discussion is provided in the section. Lastly, the section provides the main conclusions of this study and recommendations for design of modified HMA and future research directions.

## Methodology

**Fig. 1:**
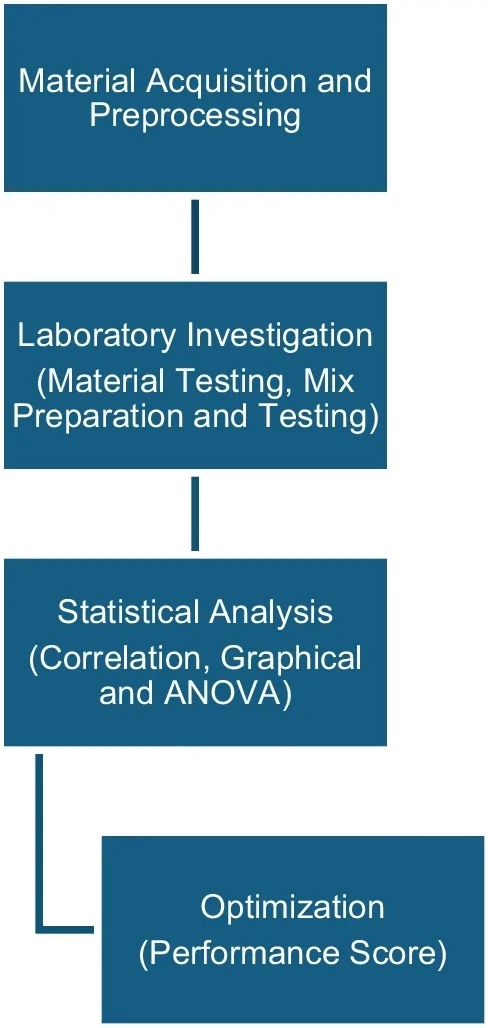
Research methodology framework

The research methodology primarily involves laboratory investigations, analysis of results using correlation and ANOVA, and determination of practical ranges using performance scores proposed in this research. The research methodology framework is shown in Fig. 1.

### Laboratory investigations

The laboratory investigation encompasses determining the general properties of materials used in the study and preparing specimens for evaluating various engineering parameters.

#### Determination of general properties of materials used in the study

Standard tests were conducted to assess the general properties of conventional materials. A total of 300 samples were prepared, with RPWA sourced from various local recyclers to evaluate the properties of locally produced recycled plastic bags in Pakistan.

After the acquisition of plastic waste, a multi-stage sorting and processing framework was adopted to maintain its consistency. It started with manual sorting through visual inspection of non-LDPE elements from the waste which was then cleaned through mild detergent getting rid of surface impurities. After which, the cleaned plastic was shredded through a high-speed rotary granulator to achieve the required particle size (1.18 to 2.36 mm) which also homogenized the particle film thicknesses. For full-scale implementation, this process would require use of intelligent sorting systems such as near-infrared (NIR) at the first stage while the technologies adopted at later stages are widely available and used in the commercial plastic recycling facilities. Apart from shredding, other mechanical properties were also determined for the RPWA which showed the bulk density within the range of 0.38 to 0.42 g/cm^3^, specific gravity of approximately 0.92, and a softening threshold of 120 °C as a result of oven-heating. These properties allow the material type to be labeled as LDPE.

The RPWA was generally rough in texture, glossy in appearance, and granular in shape and reduced to above-mentioned required particle sizes. The study aimed to identify the optimal application of RPWA in the structural layers of flexible pavement (wearing course) to enhance pavement performance. The aggregate grading was adjusted to comply with national specifications (Asphalt Institute 1992). Control samples were also prepared for comparison.

The approach of replacement by weight aligns directly with the field practices adopted for preparation of HMA on large scale. It is acknowledged that taking RPWA as a proportion of volume may result in lower quantities, but the adopted approach (by weight) is justifiable since the replacement material is a waste product in which case utilization of higher quantities facilitates the disposal of materials.

As per the nationally available and used binder type for highways, grade 60/70 asphalt was sourced from national refinery limited (NRL) which corresponded to the following properties.

- Penetration at 25 °C 60–70 dmm
- Softening point ~ 49–67 °C
- Ductility at 25 °C 51 > 100 cm
- Flash point > 230 °C (min.)
- Specific gravity ~ 1.02

Locally available aggregates were used in this study, and their grading was performed according to National Highway Authority (NHA) standards. Gradation is the primary concern of the pavement construction agency because they provide allowable aggregate gradation. The aggregate gradation employed in this research is NHA Type B for coarse gradation and fine gradation. The NHA’s gradations are shown in Table 1.

**Table 1:** NHA’s aggregate gradation requirement

Sieve designation	Percent passing by weight	Coarse	Fine	Centre
mm				
19	100	100	100	100
12.5	75–90	75	90	82.5
9.5	60–80	60	80	70
4.75	40–60	40	60	50
3.38	20–40	20	40	30
1.18	5–15	5	15	10
0.075	3–8	3	8	5.5

#### Preparation of conventional and modified asphalt mix specimens

The preparation and testing of control and recycled mix specimens are detailed in Table 2, following specified standards. To establish the optimum asphalt content for both control and recycled mixes, specimens were prepared per MS-2 (John 2003) requirements. As per this procedure, the Marshall mix design procedure was applied with asphalt contents varying between 4 and 6%, with increments of 0.5%. The procedure showed that 4.5% resulted in the highest possible stability and lowest flow while maintaining air voids close to 4%. The same process was adopted for each level of RPWA; however, it was found that the optimum content changed with the proportion of RPWA which would not allow a fair comparison across the studied range. Hence, an average value of 5% optimum content was selected for the RPWA samples.

**Table 2:** Number of test specimen prepared

S. No.	Objective of preparing test specimen	Standards	Number of specimens	
			Controlled	Modified mix
1	Optimum asphalt content determination	ASTM D1559	15	15
2	Determination of stability, flow and unit weight	ASTM D1559	50	250

The sample preparation procedure follows a multiple-step process for a surface course mixture starting with the standard aggregate gradation as per NHA Pakistan’s standards using a maximum aggregate size of 19 mm. These aggregates (RPWA and natural) were oven-dried at 105 °C to get rid of surface moisture. The asphalt binder was kept in the range of 150–155 °C, and the contents were mixed at approximately 160 °C. Before this, RPWA and natural aggregates were dry blended for 60 s using a mechanical mixer to achieve a uniform mixture to be added with the binder. The mixing with the binder was done for 3 min to have a homogeneous mixture with maximum coating of the aggregates. The compaction was done immediately after mixing using a Marshall hammer with 75 blows per face (for heavy-traffic surface course design) at a compaction temperature of 145–150 °C. The specimens were kept in the molds for 48 h to allow for sufficient cooling and stability before testing them. A total of 300 specimens of specified thickness (63.5 mm) and diameter (101.6 mm) were produced according to ASTM 1559 and the modified Marshall method (US Army Corps of Engineers 1995). Of these, 50 were control specimens, while the remaining were prepared by substituting natural aggregate with RPWA at proportions of 2.5%, 5%, 7.5%, 10%, and 15% by weight, as shown in Table 2.

#### Proposed modification in bulk specific gravity calculation of modified mix

Standard test procedures were followed to determine the specific gravities of individual materials, with the harmonic mean used to calculate the bulk specific gravity of the mix. However, the conventional equation for calculating the bulk specific gravity was adapted for mixes incorporating RPWA as an aggregate replacement. The modified equation, presented as Eq. 1, includes an additional term in both the numerator and denominator to account for RPWA.

1 $$G=\frac{P_{CA+}P_{FA}+P_{MF}+P_{RPWA}}{{\displaystyle\frac{P_{CA}}{G_{CA}}}+{\displaystyle\frac{P_{FA}}{G_{FA}}}+{\displaystyle\frac{P_{MF}}{G_{MF}}}+{\displaystyle\frac{P_{RPWA}}{G_{RPWA}}}}$$

where *G*, average bulk specific gravity of all aggregates; *P*_CA_, *P*_FA_, *P*_MF_, and *P*_RPWA_, percentages of coarse aggregate, fine aggregate, mineral filler, and recycled plastic waste aggregate in a mix, respectively; and *G*_CA_, *G*_FA_, *G*_MF_, and *G*_RPWA_, specific gravity (s) of coarse aggregate, fine aggregate, mineral filler, and recycled plastic waste aggregate.

### Performance score for RPWA replacement

Considering the multivariate nature of the experimental program and the impacts of RPWA, it was deemed essential to come up with a method to optimize the proportion of RPWA content. This was achieved through a novel performance score, calculated on the basis of important experimental parameters which are used as major constraints in the Marshall mix design process indicated by the constraints included for them. The calculation of the performance score follows a sequence of expressions shown in Eqs. 2 to 7.

2 $$Score\_Stability=\left\{1.0,\;if\;mean(Stability)\geq9.81,\;otherwise\;max(0,1-(9.81-mean(Stability))/(range(Stability)\times0.5))\right\}$$

3 $$Score\_VMA=\left\{1.0,\;if\;mean(VMA)\geq14.0,\;otherwise,max(0,1-(14.0-mean(VMA))/(range(VMA)\times0.5))\right\}$$

4 $$Score\_Flow=\left\{1.0,\;if\;.0\leq mean(Flow)\leq3.5,\;Otherwise,\;max\;(0,1-dis\tan ce/(1.5\times2))\right\},\;where\;dis\tan ce=min(\left|mean(Flow)-2.0\right|,\;\left|mean(Flow)-3.5\right|)$$

5 $$Score\_AirVoids=\left\{1.0,\;if\;4.0\leq mean(Air\_Voids)\leq7.0,\;otherwise,max(0,1-dis\tan ce/(30.\times2))\right\},\;where\;dis\tan ce=min(\left|mean(Air\_Voids)-4.0\right|,\left|mean(Air\_Voids)-7.0\right|)$$

6 $$Score_SF=(mean(S/F)-min(S/F))/(max(S/F))$$

7 $$Total\_Score\_RPWA=Score\_Stability+Score\_Flow+Score\_AirVoids+Score\_VMA+Score\_SF$$

In the above framework, Eq. 7 shows that all scores are added with equal weights. However, the calculation of each of the component incorporates the design criteria in terms of priorities. For example, air voids score has a dual penalty mechanism while stability is restricted on only one side. Hence, the component scores are designed to properly capture the essence of design guidelines and are penalized and rewarded accordingly to the extent they are close to the ideal design for Marshall method.

Therefore, the final score is devised to maximize favorable properties, such as stability, using linear scaling from worst to best performance. On the other hand, it gives a perfect score for properties set with a minimal limit. It targets range properties by assigning a perfect score if the parameter is within range and penalizing otherwise. It uses equal weighting wherein all properties contribute equally to final score. The use of normalized scale allows comparison across different measurement units. Therefore, the RPWA percentage with the highest total score can be considered “optimal” because it would provide the best balance across all performance criteria. As per the standard values for Marshall mix design provided in (John 2003), the criteria used for the performance score included the following: stability ≥ 9.81 kN, flow 2–3.5 mm, air voids 4–7%, and VMA ≥ 14%, which is shown in the above equations.

## Test results and analysis

The provided text outlines the presentation and analysis of test results for asphalt mixes, including both control and modified mixes with recycled plastic waste. Below is a summary and explanation of the key points.

In this study, direct measurements were carried out for stability, flow, and unit weight using standard test procedures outlined in references. In addition, stability-flow quotient, voids in mineral aggregate, and percent air voids were derived using formulas specified in MS-2 (John 2003).

The US Army Corps of Engineers method clearly specifies the acceptable limits for air voids and flow; hence, the samples which violated the limits for a number of parameters were discarded from the available data while those having out-of-range results for one or two parameters were kept in the analysis. Applying these criteria, 10% of the samples were excluded from the data and the remaining were used for the analysis.

**Fig. 2:**
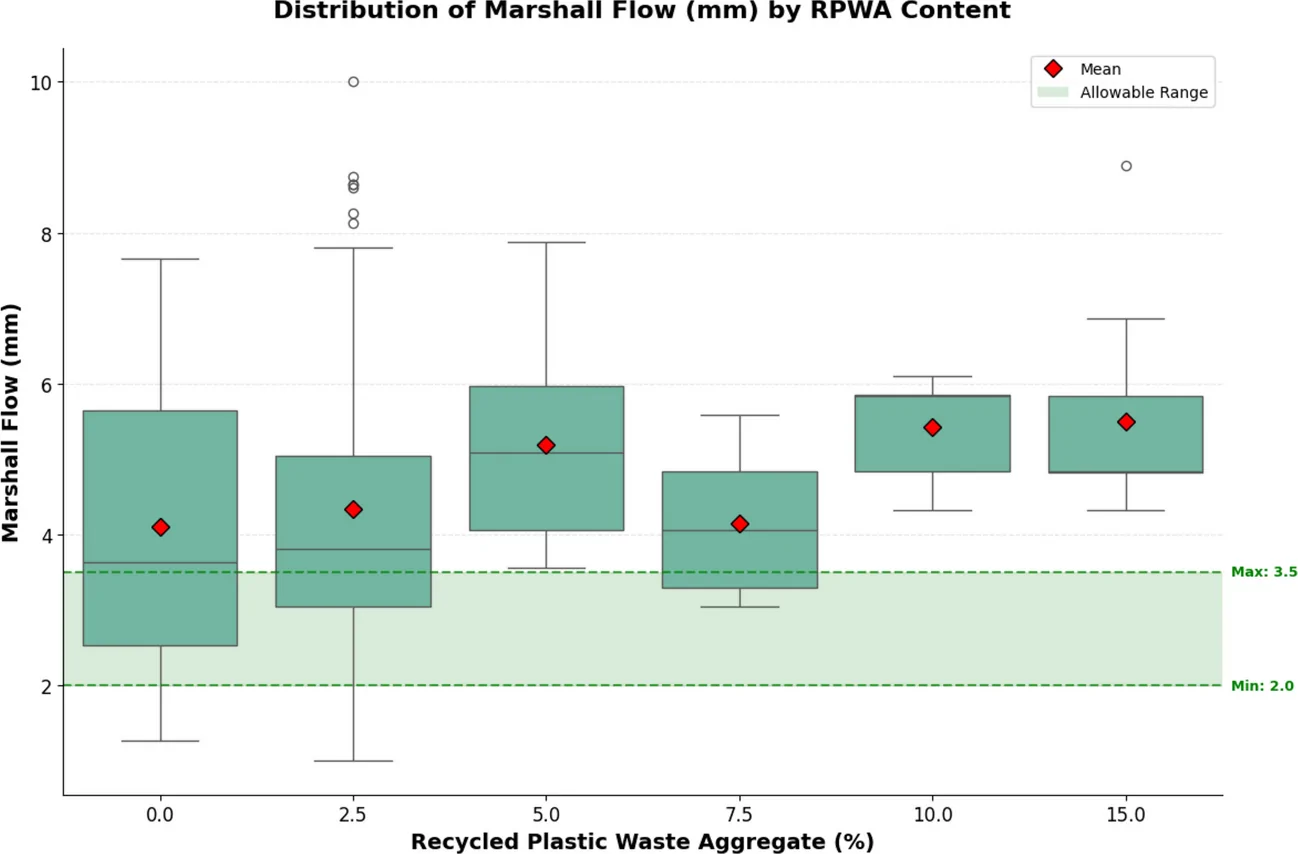
Box plots for the flow comparison at different proportions of RPWA

**Fig. 3:**
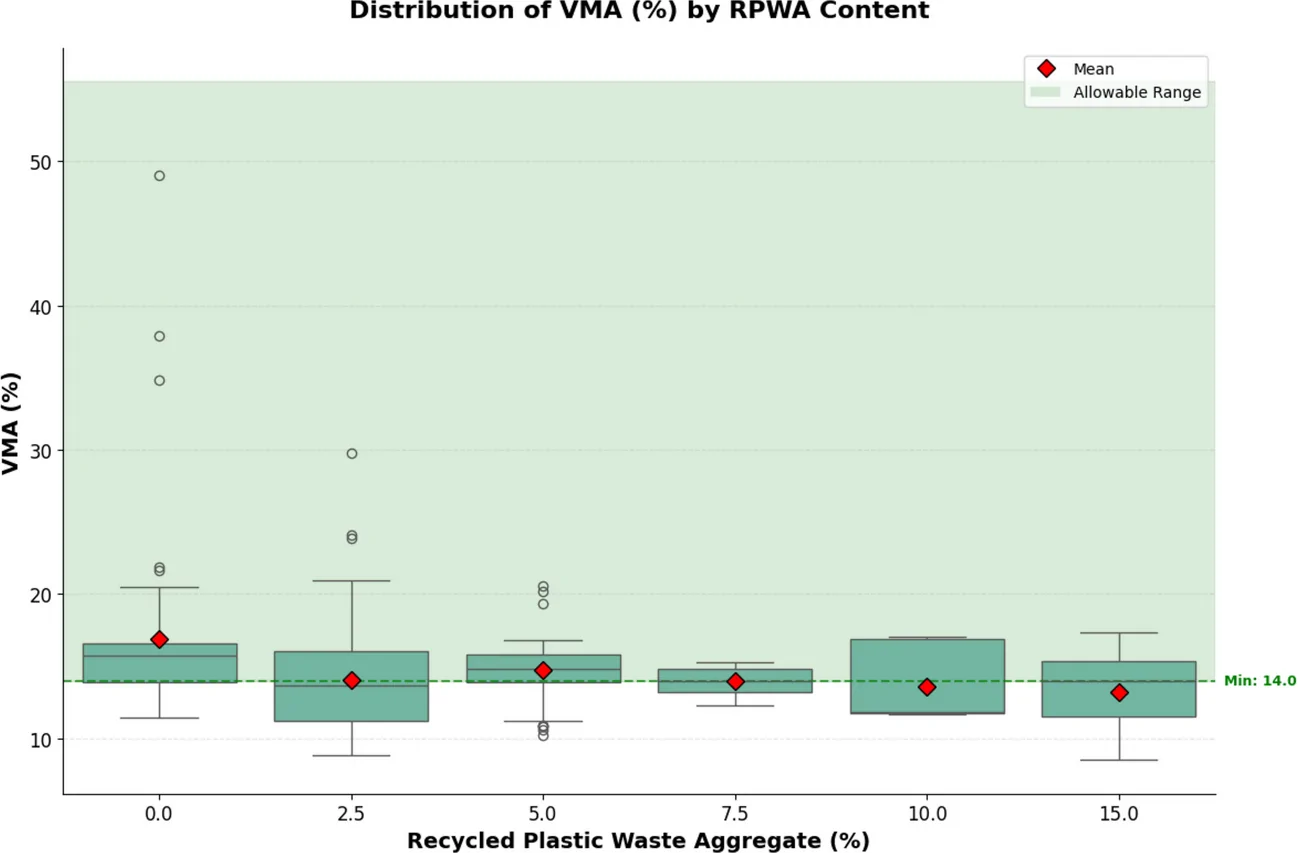
Box plots for the VMA comparison at different proportions of RPWA

**Fig. 4:**
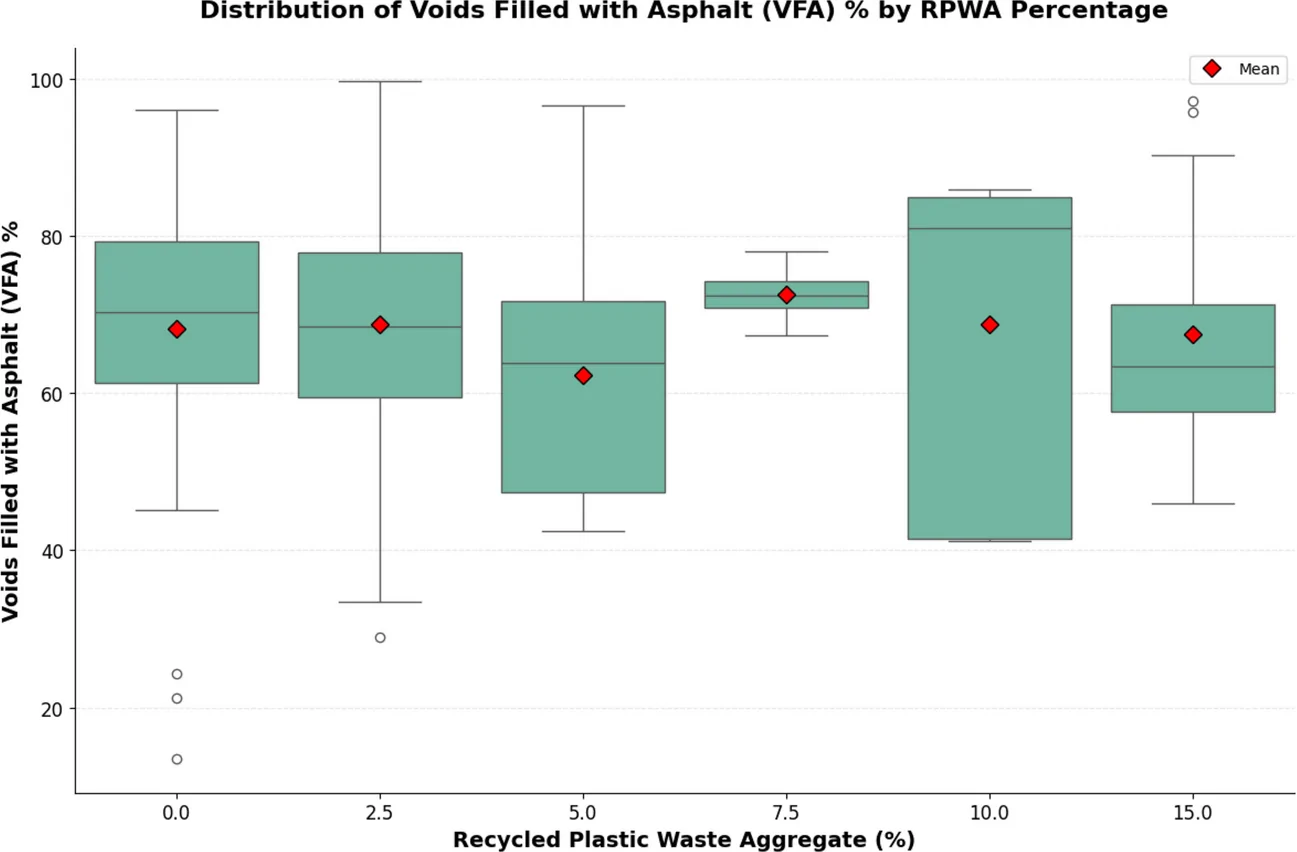
Box plots for the VFA comparison at different proportions of RPWA

**Fig. 5:**
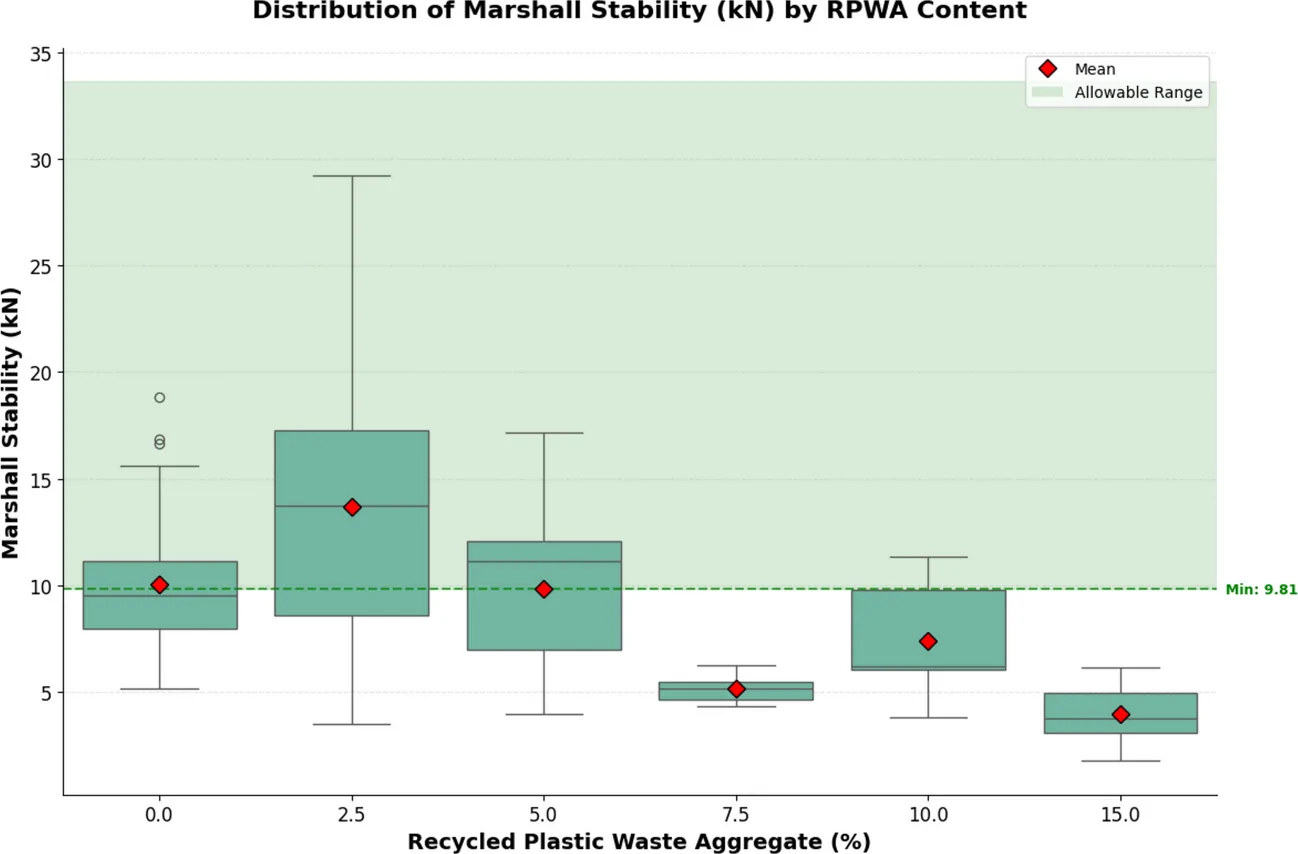
Box plots for the stability comparison at different proportions of RPWA

**Fig. 6:**
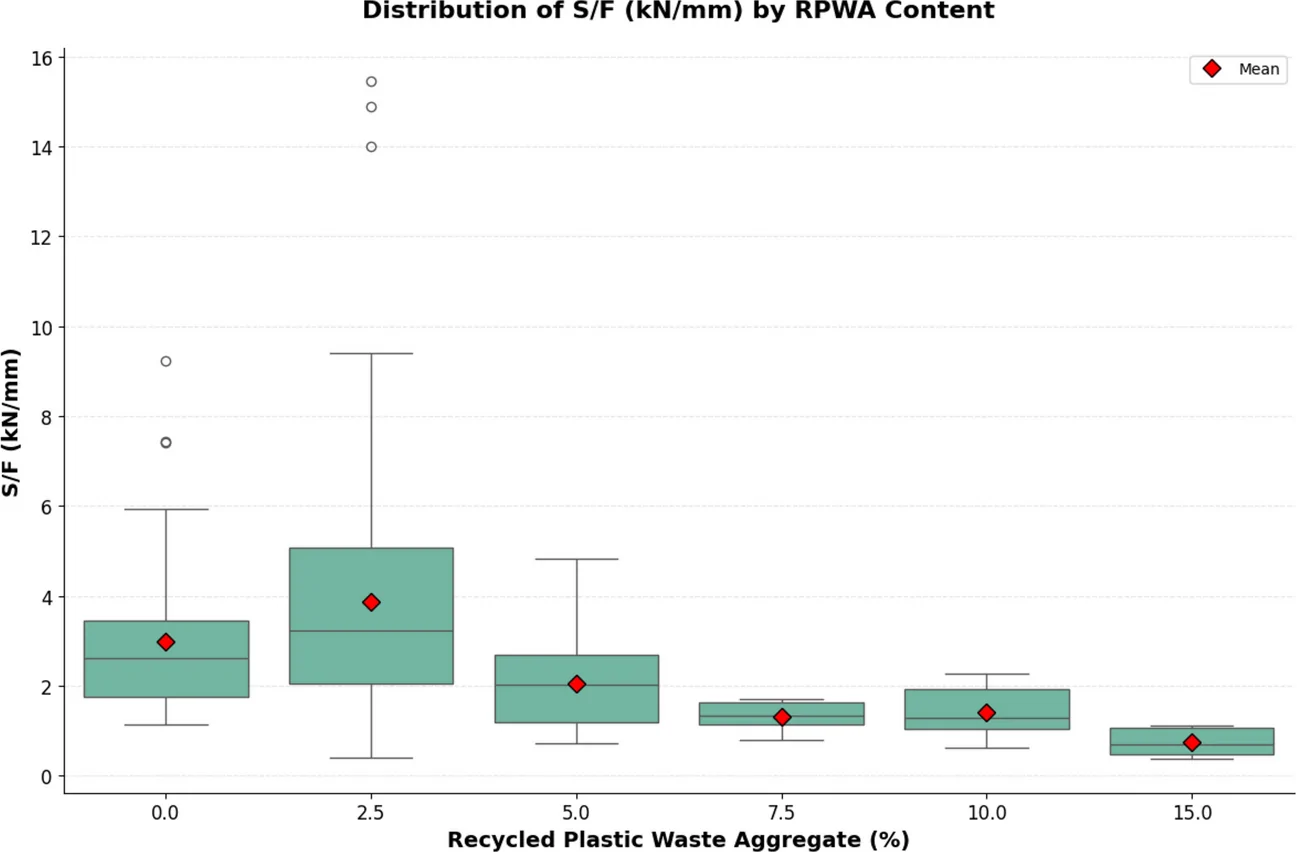
Box plots for the S/F comparison at different proportions of RPWA

**Fig. 7:**
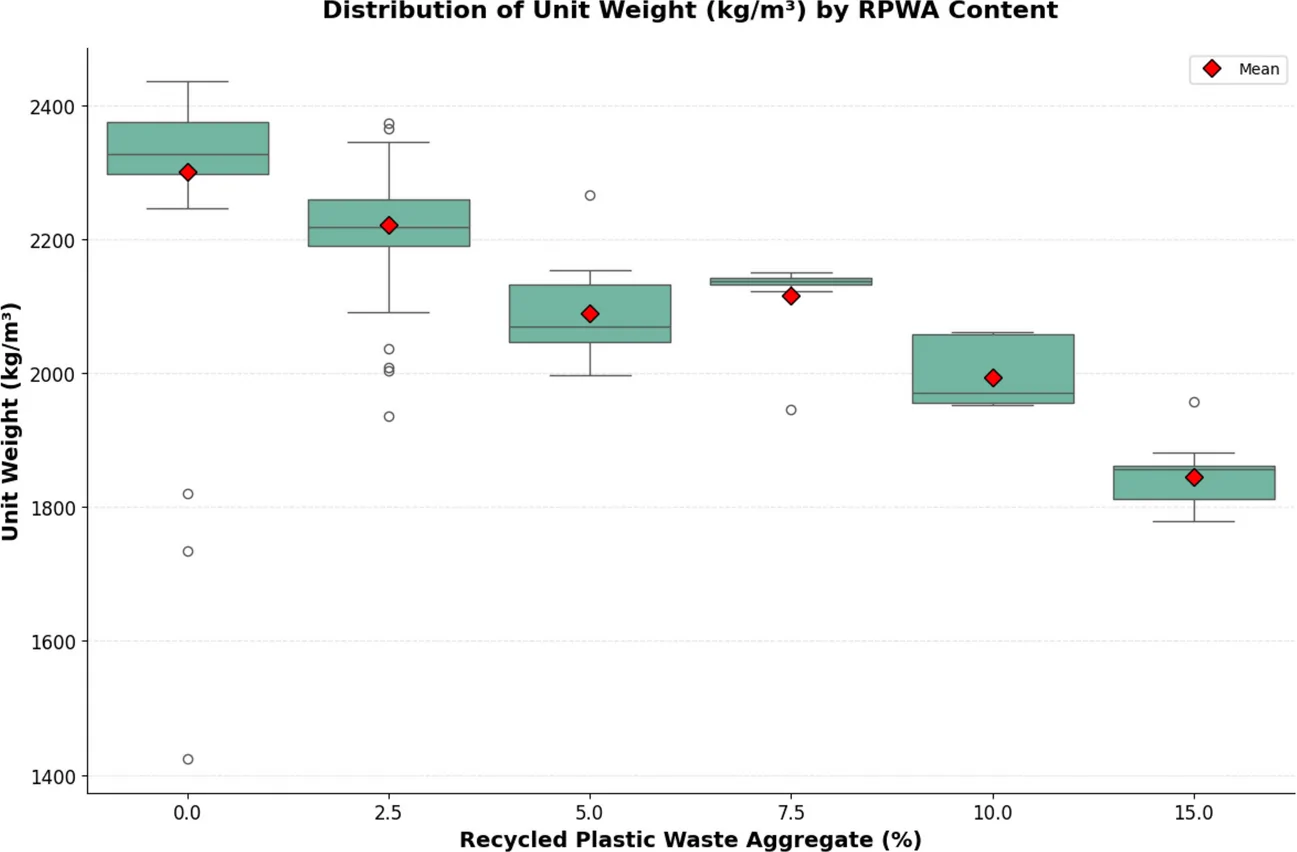
Box plots for unit weight comparison at different proportions of RPWA

The general trends for the experimental data, in Figs. 2, 3, 4, 5, 6, and 7, show that the mean Marshall Stability is above the national specification minimum. This average compliance suggests that the mix generally retains sufficient load-bearing capacity irrespective of RPWA content. The mean Marshall Flow is 4.44 mm, which exceeds the maximum acceptable limit of 3.5 mm set by the Marshall standards (2.0–3.5 mm). This is a strong indicator of the significant increase in flow caused by RPWA. This increase indicates greater plasticity or flexibility due to the softer, more deformable nature of the plastic aggregates, which practically suggests reduced resistance to rutting for these mixes. The performance of flow with regard to specific RPWA contents will be shown and discussed later.

It is also shown that the mean air voids of 5.95% and mean VMA of 14.77% both fall within the acceptable ranges of 4.0–7.0% and ≥ 14.0%, respectively. Finally, the mean unit weight was found to be 2181.40 kg/m^3^ for the samples. In conclusion, the overall summary in Table 3 suggests a largely compliant mix when considering all data points collectively. However, this compliance could be misleading because being driven by the performance of the certain RPWA content mixes (very high or very low). Hence, a deeper look at the trends for individual RPWA percentages is deemed necessary.

**Table 3:** RPWA correlation with the experimental paramaters

Parameter	Correlation_Coefficient	*p*_value	Significance
Flow	0.223	0.000613	***
Air_Voids	−0.065	0.326198	NS
VMA	−0.236	0.000287	***
VFA	−0.132	0.043573	*
Stability	−0.39	0	***
S_F	−0.336	0	***
Unit_Weight	−0.753	0	***

*p-value < 0.05, **p-value < 0.01, ***p-value < 0.0001

The correlation analysis (Table 3) provides a summary of the linear relationships between the RPWA content and physical and volumetric properties of the asphalt mixtures measured in the experimental program of this study. This analysis highlights critical dependencies between the mix properties and RPWA, consequently aiding in understanding the impact of RPWA substitution on the overall mix performance. The significant correlations in Table 3 confirm that RPWA, being lighter than natural aggregate, results in lower mix density and thus affects the volumetric properties significantly, especially flow and VMA. This trend also corresponds well with the subsequent drop in stability at higher RPWA levels.

**Fig. 8:**
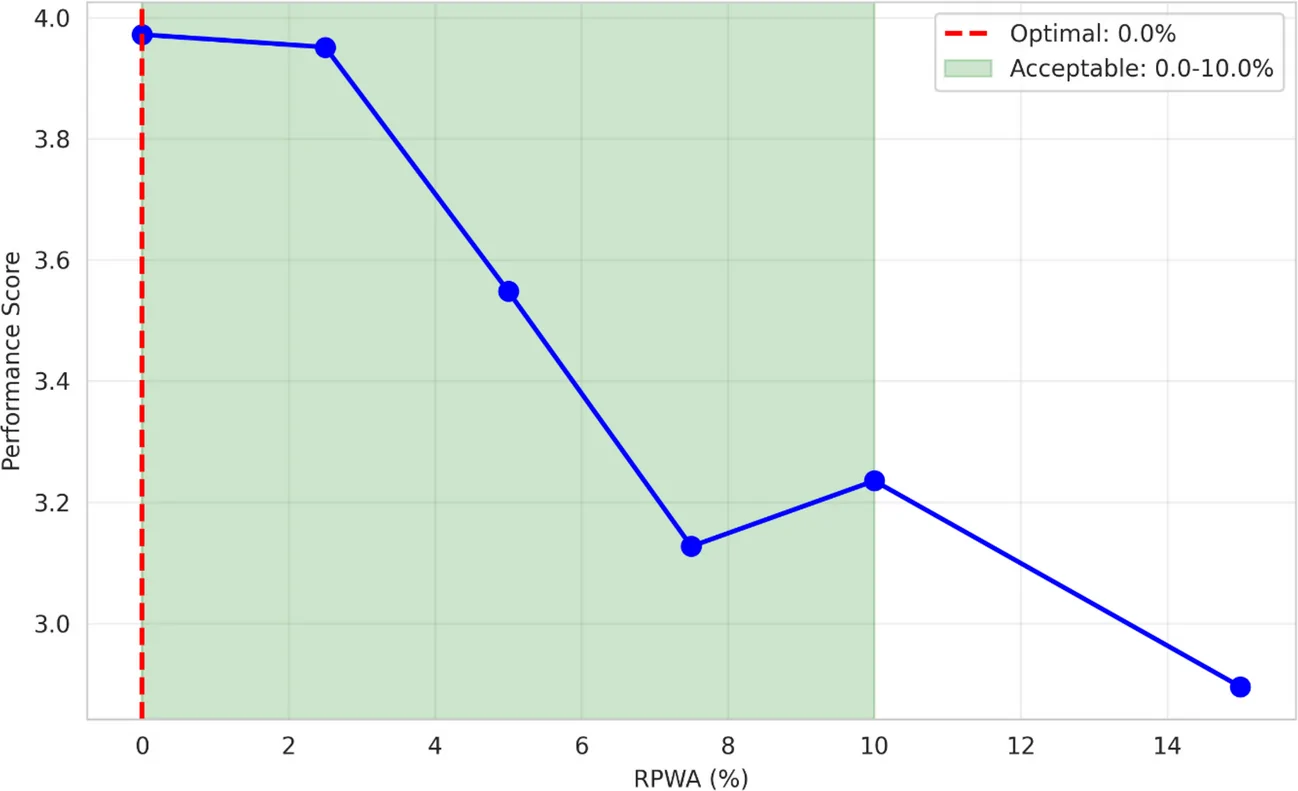
Performance score for RPWA content

**Fig. 9:**
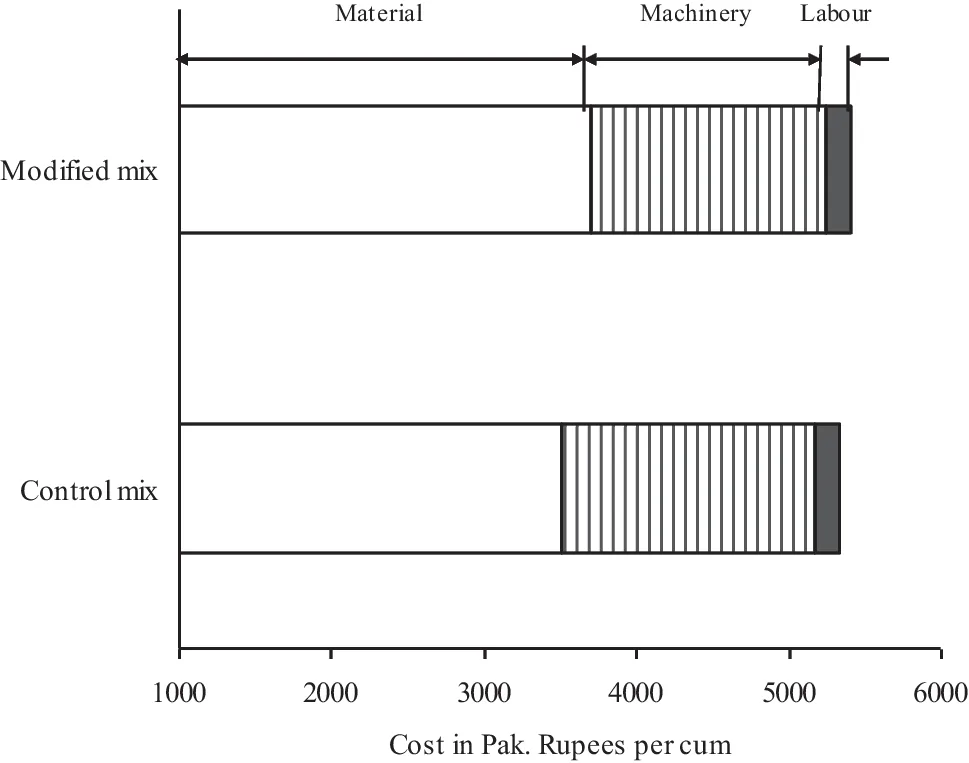
Effect of RPWA on mix preparation cost

Box plots were used to study the trends with regard to RPWA contents with the studied parameters. These box plots are presented in Figs. 2, 3, 4, 5, 6, and 7 in which mean is represented by a point estimate (red trapezoid), the box itself shows the interquartile range (IQR = 75th and 25th percentiles), the horizontal line represents the median (50th percentile), and circles represent extreme outliers. The extreme outer bars in these figures are known as whiskers after accounting for the outliers (1.5xIQR). Figure 8 represents the change in performance score (Eq. 7) with respect to change in RPWA content. Lastly, Fig. 9 presents a cost comparison for laying one cubic meter of asphalt wearing course for control and modified mixes, displayed as bar charts. ANOVA was performed to determine the significance of impact of RPWA content on the properties, which is shown in Table 4. A confidence level of 95% (5% error) was used for the ANOVA. Prior to the ANOVA, the assumptions for normality and homogeneity of variance were evaluated using Shapiro–Wilk and Levene’s test, respectively. Both assumptions were found to be valid at *p*-value > 0.05 for all parameters.

**Table 4:** ANOVA results

Parameter	Significant vs control (0%)	Effect size (vs control)
Unit weight	2.5%, 5%, 7.5%, 10%, 15%	Large (*d* = 0.722–2.928)
Stability	2.5%*, 7.5%, 15%	Medium–Large
S/F	15% only	Large (*d* = 1.480)
VMA	2.5%, 15%	Medium (*d* = 0.628–0.634)
Flow	15% only	Large (*d* = 0.809)
Air voids	None	–
VFA	None	–

The mean flow value shows a general increasing trend as RPWA content increases from 0.0 to 15.0% in Fig. 2. The peak flow values are observed around 10.0 to 15.0% RPWA. On the other hand, the mean flow at 7.5% RPWA is the lowest among the modified mixes.

The mean VMA value generally decreases with increasing RPWA content as shown in Fig. 3. The 7.5% and higher RPWA content show the lower mean VMA. Moreover, VFA shows high variability in the samples at higher RPWA proportions. This is represented by the difference between median (centerline of the box) and the mean in Fig. 4. This is also indicative of the nonlinear trend of VMA across different proportions of RPWA, which is highly aggravated at 15% RPWA content. The highest VMA is observed at RPWA% of 2.5 and 5.0. This suggests that a lower RPWA% allows for greater binder absorption in the voids. On the other hand, low values at higher RPWA% (7.5–15%) may indicate a change in aggregate packing structure due to the displacement of binder by plastic aggregates affecting the void filling efficiency.

The stability also shows a nonlinear trend with respect to RPWA content, with the peak value at 2.5% in Fig. 5. This gain at the lower substitution level could be attributed to the plastic acting as a modifier and filler providing better interlocking of aggregates and internal adhesion between the mix components. There is a sharp decline in stability when the RPWA content increases more than 2.5% indicating the dominating effect of softening of plastic aggregates over the sample. The results attained at 15% RPWA content go below the standard levels of 9.8 kN.

The S/F ratio is a critical performance indicator for HMA, representing stiffness index mix which is directly proportional with its rutting resistance. The index shows a declining trend with respect to RPWA content in Fig. 6. The peak stiffness is attained at 2.5% content representing the stiffest of mixes. The decline is consistent beyond 2.5% representing a progressive loss of internal friction and cohesion. Hence, high contents of RPWA are highly susceptible to premature rutting failures, especially when subjected to higher temperatures. The increasing spread of data towards the higher RPWA contents shows an increase in inconsistency of results.

The trend of unit weight with respect to RPWA is declining with highest value at 0% content and lowest at 15% as shown in Fig. 7. This is directly related to the lower specific gravity/density of RPWA as compared to natural aggregates. The spread data show better consistency depicting stability in the samples with regard to unit weight measurements.

Table 4 shows that five of the six parameters studied in this research are found to be significantly impacted by RPWA. These include flow, VMA, VFA, stability, S/F, and unit weight, with (*p*-value < 0.001). Unit weight, stability, and S/F show large effect sizes of RPWA (*η*^2^ > 0.3) causing significant difference from the control for these mechanical properties. On the other, the effect size is medium for VMA and flow (*η*^2^ between 0.71 and 0.83) suggesting moderate influence. The insignificant impacts on air voids and VFA indicates the structural soundness of the mix in terms of void characteristics. In all cases, 15% RPWA was significantly different from variables which had significant differences across RPWA values confirming that higher replacement levels are likely to change the mechanical properties. The Marshall mix design process does have a strict range of air voids for an acceptable design. Therefore, it was still used in the calculations of the performance scores despite lack of relationship between it and RPWA content.

As mentioned above, a performance score (Eq. 7) has been proposed in this research for optimizing the range of RPWA content in order to cater the multidimensional criteria of HMA process. Based on this score, Fig. 8 that the 2.5% RPWA mix achieves the second-highest score, after the control samples (at 0% RPWA), aligning with earlier findings. In total, 2.5% content provides the best balance between strength (stability) and deformation (flow and VMA) with the incorporation of sustainability (utilizing waste material) without losing much on the design aspect. The sharp decline in the score after 2.5% content shows the sensitivity of the HMA samples to higher values of RPWA content. Beyond that, the acceptable range in the figure goes up to 10% showing the samples which can fulfil the Marshall mix design limits not necessarily providing the best possible parameters.

Figure 9 shows the comparison of preliminary costs of laying out 1 m^3^ of HMA with standard and modified mixes with RPWA. The unit rate for the standard mix was taken from the market at PKR5325/m^3^ while following assumptions were used to estimate the cost of the modified mix. The collection and sorting rate was taken at PKR15/m^3^, which is derived from the assumed rate of 88–100 per ton for the sorted plastic waste with 15% replacement of aggregates. The washing and shredding costs were ascertained from the reported costs in the literature amounting to $4–6 per ton which transforms to PKR40/m^3^. It is reasonable to transfer these costs for such mechanical processes. Incremental labor costs were taken at approximately 10% and an additional 5% was added to the cost of machinery to account for increased mixing time and wear from abrasive plastic materials.

Following this process, the modified mix cost came out to be slightly higher (PKR5400/m^3^) compared to the control mix. As explained above, the material cost has the highest proportion in both cases which is also the main reason for higher cost of modified mix as it involves preprocessing of waste plastic. Moreover, the machinery cost is also higher for the modified mix due to additional handling and mixing requirements. The labor costs are the lowest and constant for both types of mixes. Having taken all these aspects into consideration, modified RPWA mix has a marginal increase in cost of the mix but has the potential to provide additional benefits in terms of mitigating environmental impacts of waste plastic when mixed optimally to meet the design criteria.

## Discussion

The results of this study clearly demonstrate a significant impact of RPWA on HMA properties and provide a distinct optimal dosage of RPWA. It was observed that a small amount of RPWA (2.5%) acts as a beneficial modifier, providing a balanced mix with Marshall Stability going beyond the control mix. Hence, there appears to be a “phase change” of RPWA before and after the threshold of 2.5% content value which needs further validation with tests related to coating behavior, aggregate interlock, binder absorption, plastic deformation, particle shape, and density. The plastic particles act as a high-performance filler and binder modifier when in lower proportions, enhancing stability by improving cohesion and aggregate interlock. Beyond the threshold, these particles become a soft, non-load-bearing aggregate that disrupts the stone-on-stone contact of the mineral skeleton. This aligns with studies (Joni and AL-Rubaie 2022; Khan et al. 2019) that report improved stability and rutting resistance at low plastic content. The trend is further illustrated by the peaks in stability and S/F boxplots, in Figs. 6 and 7, respectively. Having said that, the current study advances the current knowledge by showing the non-linear nature of these trends.

The impacts of RPWA on VMA and unit weight are some of the less discussed aspects in the literature. Moreover, the VMA is also found to be the lowest between the range of 2.5–7.5% RPWA content indicating the functionality of melted plastic in coating the aggregates and filling the gap. Several studies have reported shifts in void characteristics with the introduction of a new material in HMA because of its unique absorption and coating characteristics (White and Hall 2022; Khan et al. 2019). Although the direction and magnitude of this impact can vary for different materials. However, the higher VMA at higher RPWA contents, along with a consistently declining trend in unit weight, indicates a change in aggregate packing structure and possible displacement of binder. The possible reasons include plastic’s low density and inability to compact like a mineral aggregate. This is a key contribution towards understanding the failure mechanism of high RPWA content mixes.

The impacts of RPWA contents on flow and stability parameters show that the modification reduces the elastic recovery of the mix. This is a widely reported and expected consequence (Abuaddous et al. 2021). The increasing flow values indicate that the shifting of mix towards a more plastic state increasing its susceptibility to rutting. ANOVA confirms the impact of RPWA on the mix properties while the performance score provides the optimum point (2.5%) at which the phase of RPWA appears to change from reinforcement to contaminant. This simplistic analysis is found to be effective in identifying a precise optimal range of RPWA content advancing the research beyond the mere determination of single optimum value.

The preliminary unit cost comparison of the control and modified sample shows a marginal increase for the latter which is due to the material and machinery costs due to preprocessing of plastic, and its handling and processing. Against this marginal increase, the modified mix (at the 2.5% content) provides the engineering benefits of highest load bearing capacity and environmental benefits of mitigating impacts of plastic waste handling and disposal. However, the cost comparison is limited to the placement cost, and a complete lifecycle cost analysis (LCCA) will provide a clearer picture.

## Limitations

There were certain limitations to this study, including use of specific type of plastic (LDPE), not implementing full-scale field performance measurement, focusing on deploying Marshall method parameters while not including other critical performance indicators such as fatigue, tensile strength, long-term aging, and lastly, not performing a full LCCA. Moreover, lack of moisture evaluation also hindered evaluation of binder adhesion, aggregate coating, and moisture resistance. The practical recommendation of using RPWA in roads is conditional on the verification of these parameters. Due to these limitations the following recommendations are made for future research. Investigations should be done with smaller increments in RPWA content between 0 and 5% to refine the optimal dosage. Advanced performance testing should be employed to evaluate rutting resistance, fatigue life, moisture damage (TSR), and thermal properties of the optimal mix. The impacts of using other common waste plastics (e.g., HDPE, PP, PET) and mixed-plastic streams on HMA should be explored. Field validation should be conducted with pilot road projects to monitor the long-term field performance and durability of RPWA-modified asphalt. Finally, comprehensive LCCA and environmental assessment models should be developed to include collection, sorting, preprocessing, and long-term maintenance costs. The known environmental concerns in this regard include microplastic release, leaching, fume emissions during mixing, and end-of-life milling or recycling which need specific attention for the complete assessment of environmental benefits of plastic waste disposal in the proposed manner. Despite these limitations, this research still provides a viable solution to reducing plastic waste and conservation of natural materials through the optimum use of recycled plastic in large-scale construction.

## Conclusions

This study deals with the utilization of waste plastic which is one of the most concerning waste materials of modern times, especially in Pakistan. It aims to analyze the impacts of adding RPWA in HMA mixes using a wide range of variables by employing Marshall mix design procedure. By doing so, it also underscores the possibility of conserving natural materials (aggregates) with the use of recycled waste. The results of this research show the magnitude and nature of RPWA impacts, provide valuable insights about the internal changes caused by it and recommend optimum range for an acceptable design with the best value for a balanced mix. A data-driven framework is employed with the novel performance score to handle the multivariate selection mechanism.

Incorporation of RPWA, sourced from LDPE polyethylene bags, as a partial aggregate substitute in HMA was found to be both technically feasible and environmentally beneficial, within specific limits. The main conclusions of this study are outlined below:

- The replacement of 2.5% provided the best strength among all the modified samples, after which it showed a sharp decline while replacement of 15% resulted in lower than standard value (9.81 kN).
- Marshall flow was positively correlated with RPWA with higher proportions resulting in flow values exceeding 3.5 mm.
- Consequently, S/F peaked at RPWA proportion of 2.5% and then declined consistently after that.
- Air voids and VMA remained within the specified limits across all levels of RPWA. The observation was confirmed by ANOVA which showed insignificant impact of RPWA on these parameters.
- RPWA had a statistically significant effect on five of the seven parameters studied: Flow, VMA, VFA, stability, S/F, and unit weight (*p* < 0.01), with effect sizes ranging from medium to large.
- Post-hoc analysis showed a consistent deviation of properties of 15% RPWA levels from the control samples.
- Based on the proposed performance score, 2.5% RPWA level provided the highest score among the modified samples which was still lower than control samples. This indicates a trade-off between sustainability and design scores.
- The preliminary cost estimates show a negligible escalation of 1.4% in the preparation costs of modified samples compared to the control samples.

Based on these results, RPWA can be used to enhance pavement performance, but strict quality control on batching is crucial due to the narrow optimal range. The findings of this study pave the way towards a high-volume, sustainable disposal pathway for low-value plastic film waste. However, its applications would be more suitable for low to medium-traffic roads initially, as per the required performance profile. In such cases, the modest material cost increase, shown in the initial cost estimates in this study, is justified by the dual benefit of significant positive environmental impact with negligible loss of design properties.

## Data Availability

The manuscript has data included as electronic supplementary material.
